# Advances in Chimeric Antigen Receptor (CAR) T-Cell Therapies for the Treatment of Primary Brain Tumors

**DOI:** 10.3390/antib11020031

**Published:** 2022-04-27

**Authors:** Christopher W. Mount, Luis Nicolas Gonzalez Castro

**Affiliations:** 1 Department of Pathology, Massachusetts General Hospital, Boston, MA 02114, USA; cmount1@partners.org; 2 Center for Neuro-Oncology, Dana-Farber Cancer Institute, Boston, MA 02115, USA

**Keywords:** primary brain tumors, glioblastoma, diffuse midline glioma, H3 K27M-mutant, chimeric antigen receptor (CAR) T-cell therapy, adoptive cell therapy

## Abstract

Immunotherapy has revolutionized the care of cancer patients. A diverse set of strategies to overcome cancer immunosuppression and enhance the tumor-directed immune response are in clinical use, but have not achieved transformative benefits for brain tumor patients. Adoptive cell therapies, which employ a patient’s own immune cells to generate directed anti-tumor activity, are emerging technologies that hold promise to improve the treatment of primary brain tumors in children and adults. Here, we review recent advances in chimeric antigen receptor (CAR) T-cell therapies for the treatment of aggressive primary brain tumors, including glioblastoma and diffuse midline glioma, H3 K27M-mutant. We highlight current approaches, discuss encouraging investigational data, and describe key challenges in the development and implementation of these types of therapies in the neuro-oncology setting.

## 1. Introduction

High-grade gliomas are the most aggressive primary brain tumors in children and adults. Despite groundbreaking advances in our understanding of the molecular origins and clinicopathologic behavior of these tumors, clinical outcomes remain dismal for pediatric and adult patients alike. In adults, a newly-diagnosed glioblastoma (GBM) patient currently faces a median overall survival time of 15–18 months, with a 5-year survival rate of less than 10% [1]. In pediatric high-grade gliomas, the outcomes are similarly poor [2]. While molecular tumor characterization has helped inform prognosis and resulted in substantial refinements to the diagnostic framework for these diseases [3], breakthrough therapies remain elusive.

**Figure 1:**
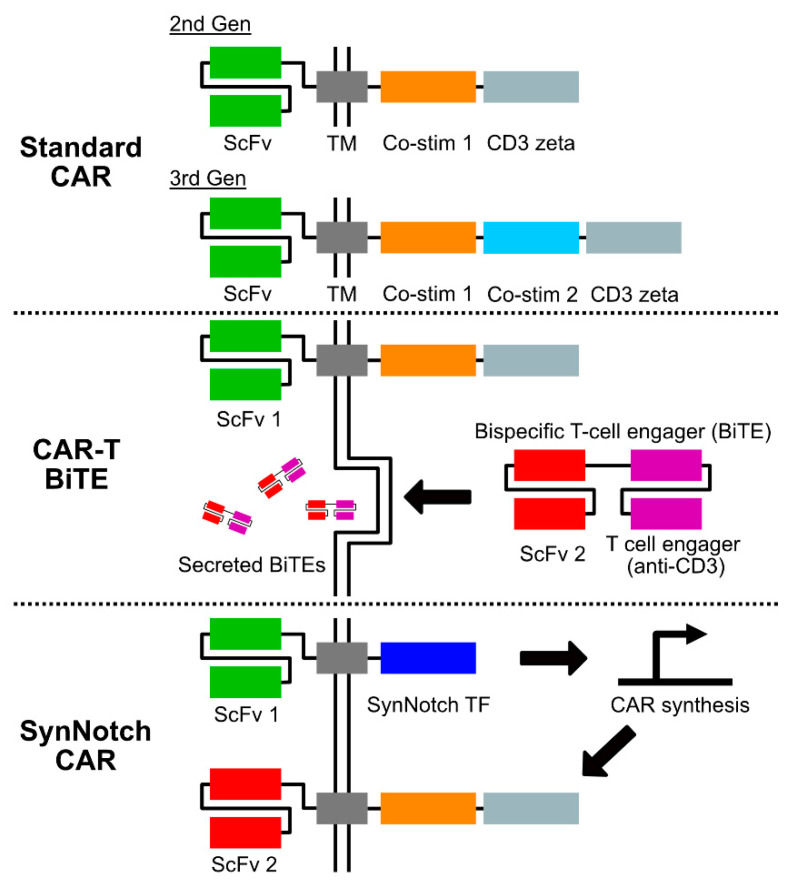
Chimeric antigen receptor (CAR) construct strategies under investigation in gliomas. Standard CAR designs under clinical investigation in GBM include both second and third generation constructs, which differ in the number of intracellular costimulatory domains. Multiple costimulatory domains are currently under investigation, with CD28 and 4-1BB most commonly used to date [6]. Combined CAR-T and bispecific T-cell engager (BiTE) constructs are an emerging strategy in which T-cells are engineered to express both a CAR and a secreted bispecific molecule with specificity to both a second tumor-targeting antigen and a surface T-cell molecule such as CD3 [9]. SynNotch CARs function as a synthetic logical AND gate [10]. Engagement of a tumor antigen target induces intracellular T-cell signaling via a synthetic Notch construct. This “priming” step induces the synthesis of a standard CAR molecule, which when expressed on the T-cell surface enables cytotoxic activity. Abbreviations are as follows: ScFv—single chain variable fragment; TM—transmembrane domain (may include hinge region); Co-stim—costimulatory domain.

In the face of these challenges, there remains hope that advances from the burgeoning field of cancer immunotherapy might help change the course of these devastating illnesses. Disappointing results from early trials of immune checkpoint inhibition in high-grade gliomas have focused interest on alternative or combinatorial immunotherapy strategies [4,5]. Adoptive cell therapies are one such strategy, in which a patient’s cytotoxic immune cells are engineered ex vivo to target tumor antigens and then re-infused. Chimeric antigen receptor (CAR) T-cell therapy represents one of the more scientifically and clinically mature approaches within this category. The CAR molecule is a bioengineered construct joining an extracellular targeting domain, most commonly derived from an antibody’s single-chain variable fragment (scFv), with intracellular T-cell activation and co-stimulation domains. In particular, the incorporation of costimulation domains—most commonly CD28 and 4-1BB—are critical to CAR T-cell function, and the arrangement of these domains is a defining feature of current CAR structures (see Figure 1) [6]. The converging forces of advances in immunobiology, increasing ease of synthetic DNA manufacture, and favorable clinical responses in different hematologic malignancies have resulted in an explosion of the CAR T-cell ecosystem [7,8]. The result is a battery of novel, promising therapies that are increasingly being evaluated in early-stage neuro-oncology clinical trials. Here, we review salient developments in CAR T-cell therapies for glioblastoma and pediatric high-grade gliomas and highlight the ongoing challenges and opportunities for the field.

## 2. Glioblastoma

Adoptive T-cell immunotherapy efforts in glioblastoma span decades, with initial approaches simply stimulating a patient’s peripheral blood lymphocytes ex vivo and injecting them into the tumor bed after resection [11]. The use of CARs to direct lymphocyte activity against GBM antigens is a more recent and promising development [12]. Interleukin-13 receptor alpha 2 (IL13Ra2) has emerged as one such CAR T-cell target in GBM, being present in a substantial fraction of tumors with minimal background expression [13]. Initial human trials by Brown and colleagues administering a first-generation anti-IL13Ra2 CAR T-cell into the resection cavity of GBMs demonstrated the therapy was well tolerated over multiple doses and led to responses in two out of the three patients treated [13]. Follow-up studies in one patient suggested downregulation of the IL13Ra2 target antigen after therapy, potentially indicating antigen-dependent selective pressures [13]. These early feasibility studies prompted the optimization of a second-generation, 4-1-BB costimulated construct [14]. These efforts crystallized in 2016, when a patient with recurrent GBM treated with an IL13Ra2-targeting CAR T-cell experienced the regression of all intracranial and spinal tumors on imaging and remarkable clinical improvement [15]. This complete response was sustained for 7.5 months, although the tumor did eventually recur at new sites [15]. Nevertheless, this remarkable response encouraged new interest in this approach, and the ongoing clinical investigation of IL13Ra2-targeting CAR T-cells is underway (see Table 1).

The epidermal growth factor receptor (EGFR) represents another target antigen of interest in GBM. The widespread expression of EGFR in multiple human tissues limits the potential for targeting the wild-type receptor [16]. However, approximately 20% of GBMs have been found to express a transcriptional variant—EGFRvIII—which has been characterized as a putative tumor-specific variant [17,18]. Building upon previous efforts to target this variant, several groups have developed EGFRvIII-targeting CAR T-cell platforms [19,20,21]. O’Rourke and colleagues reported a series of 10 GBM patients treated in a pilot study with a humanized EGFRvIII-targeting scFv, 4-1BB-costimulated CAR T-cell [19]. They demonstrated the feasible cell manufacturing and clinical safety of intravenous dosing, but no objective radiographic responses were observed [19]. One patient did demonstrate stable disease at 18 months—longer than anticipated for progressive multifocal GBM, where survival ranges in the order of 6 to 8 weeks [19]. Subsequently, Goff and colleagues reported a series of 18 GBM patients treated with an EGFRvIII-targeting, third-generation CAR T-cell incorporating both CD28 and 4-1BB costimulation [20]. While the study did demonstrate successful cell manufacturing and administration that resulted in persistent engraftment detectable in peripheral blood by quantitative polymerase chain reaction (qPCR), no radiographic responses were observed [20]. Additionally, the patient treated at the highest dose level on this escalation-based protocol developed acute respiratory failure shortly after CAR T-cell administration, leading to rapid and treatment-refractory clinical deterioration, with the patient expiring four hours after the infusion was completed. Post-mortem studies identified significant pulmonary edema [20].

Human epidermal growth factor receptor 2 (HER2)-targeting CAR T-cells have also been studied in GBM, after studies demonstrated frequent HER2 expression in this and other brain tumors [22,23]. Preclinical studies led by Ahmed and colleagues demonstrated that HER2 CAR T-cells could induce GBM regression in mouse models [24]. Initial human trials of a third-generation HER2 CAR T-cell combining CD28 and 4-1BB costimulatory domains in patients with metastatic cancer were marred by acute, lethal toxicity in a patient with metastatic colon cancer, which may have resulted from the relatively large cell infusion dose [25]. Subsequent trials of a CD28-only costimulated HER2 CAR T-cell in HER2+ sarcoma patients were free of such toxicity events, but showed low therapeutic cell persistence [26]. Building on evidence that Epstein–Barr virus (EBV)-specific cytotoxic T-lymphocytes exhibited superior persistence when used to generated CAR T-cells in other settings [27], Ahmed and colleagues evaluated HER2 CAR-modified virus-specific T-cells in GBM patients [28]. Using a second-generation construct with CD28 costimulation, HER2-CAR T-cells were tolerated in all 17 patients treated with no dose-limiting toxicities. Both adults (nine patients) and children (seven patients) were treated in this study. The responses were mixed, with imaging showing one patient with partial response and seven patients with stable disease ranging from 8 weeks to 29 months. The median overall survival from the time of infusion was approximately 11 months. While cell expansion was not seen peripherally, persistent HER2 CAR T-cells were detected in the blood up to 1 year from treatment.

Up to now, CAR T-cell therapy trials in GBM have led to limited clinical and radiographic responses, but have advanced our understanding of the development and deployment of these therapies, as well as the diagnosis and management of potential complications. Relatively few antigens have been targeted thus far in the clinical setting, and there remains no overarching consensus on which antigen(s) are the most promising targets to pursue in the relatively small study populations available for study enrollment [29]. While preclinical work continues to elucidate both new targets and novel targeting strategies, several additional CAR T-cell trials are underway in GBM (see Table 1).

## 3. Pediatric Brain Tumors

Given the similarly dismal clinical outcomes for high-grade pediatric brain tumor patients, CAR T-cell therapies also hold great promise in the realm of pediatric neuro-oncology. Expanding upon the aforementioned trials of HER2 CAR T-cells in pediatric patients, Vitanza and colleagues are leading efforts to evaluate this approach in pediatric medulloblastoma patients [30]. Preclinical studies by this group showed that the spacer length optimization of a HER2 4-1BB construct was critical to efficacy in mouse medulloblastoma xenograft models. In the clinical setting, the initial three patients from the BrainChild-01 (NCT03500991) trial reported by this group tolerated repeated locoregional administration of this optimized HER2 CAR T-cell product. While the blood–brain barrier has limited the efficacy of other small molecule and biologic therapies, its role in CAR T-cell therapies remains unclear. While in some preclinical systems, peripherally-administered CAR T-cells clearly show the ability to infiltrate the CNS and eliminate engrafted tumors [31], several preclinical studies have now suggested the increased efficacy of the locoregional administration of CAR T-cells for brain tumors compared to systemic intravenous dosing [32,33]. In the BrainChild-01 trial, the authors demonstrated the clinical safety of this locoregional approach with both intracavitary and intraventricular dosing strategies [30]. Moreover, multiple rounds of locoregional administration were both feasible and tolerated. Imaging evidence of treatment-associated peritumoral edema and increased concentrations of CSF cytokines, including CXCL10 and CCL2, was consistent with inflammation in the setting of CAR T-cell activity. Additional follow-up and expanded cohorts will be necessary to further assess efficacy.

As brain tumor classifications have become more refined [3], gliomas characterized by a histone 3 K27M mutation (H3 K27M gliomas) have been recognized as a disease with particularly poor prognosis that primarily arises in children [34]. Surface antigen screening of patient-derived H3 K27M glioma cultures recently identified the disialoganglioside GD2 as a highly expressed surface target on these tumors [31]. Preclinical studies of a 4-1BB costimulated second-generation GD2-targeting CAR T-cell demonstrated dramatic antitumor efficacy, with the ability to eradicate xenografts in mouse models [31]. These tumors arise in midline brain structures, including the brainstem, spinal cord, and thalamus, all of which are particularly sensitive areas to inflammation-associated edema. In mouse models, toxicity during the peak phase of antitumor activity appeared to correlate with the location of tumor xenografts, highlighting the potential danger of using CAR T-cell therapies for tumors in sensitive neurologic sites [31]. With this in mind, initial clinical studies of GD2 CAR T-cells in H3 K27M glioma patients were designed with the aim of developing principles of neurocritical care management with this new therapy [35]. In their report of the first four patients treated with this therapy, Majzner and colleagues highlighted the benefit of this careful incorporation of neurocritical care precautions [35]. Three of four treated patients showed radiographic improvement, including one patient with a >90% reduction in the volume of a spinal cord tumor. Critically, clinical benefit was seen alongside radiographic changes, including improved cranial nerve, gross, and fine motor function. In this diffusely infiltrative tumor, this represents compelling evidence that the T-cell-mediated destruction of an infiltrating tumor is compatible with at least partial recovery of neurologic function. The authors also demonstrated that anticipated treatment-related neurologic symptoms, which they termed tumor inflammation-associated neurotoxicity (TIAN), could be safely managed with multimodal therapy [35]. Note that the clinical presentation of neurotoxicity in these patients is related to specific tumor inflammation and distinct from the neurotoxicity described in patients receiving CAR T-cell therapy for hematologic malignancies [36,37]. While responses in these patients were heterogeneous, the experience from this clinical trial is shaping ongoing studies that have great promise an effective therapy in this devastating disease.

## 4. Challenges and Opportunities

While there is great potential for CAR T-cell therapies to become an important component of future neuro-oncology care, an objective evaluation of the trials to date indicates clinical responses have been mixed, at best [12,38]. Thus, it may be tempting to label these therapies as only marginally effective. However, there are important reasons for optimism at this early stage. It is important to note that our understanding and exploration of potential targets in gliomas is primitive compared to the setting of hematologic malignancies, where the surface antigen profile of tumors and normal hematopoietic lineages has been studied intensively for decades and is routinely used in clinical settings. Therefore, new CAR T-cell programs for gliomas have largely arisen from those designed to target major antigens in other diseases, and which are found to be expressed to some extent in gliomas [12]. A nuanced understanding of the landscape of antigen expression in gliomas is lacking. Recent comprehensive characterizations of gliomas at the single-cell level are expanding our ability to understand the heterogeneity of the transcriptional landscape of gliomas, and the complex interactions between malignant cells and the tumor microenvironment [39,40,41,42]—findings that may aid in the selection of future target antigens. Validation of these targets in the clinical setting is an ongoing challenge that will require active collaboration between multidisciplinary clinical teams and laboratory researchers to perform most effectively.

When treated with CAR T-cells, the tendency of tumors to downregulate or otherwise lose expression of a target antigen represents another major challenge for all CAR T-cell therapies, including those directed against gliomas. In hematologic malignancies, this is a well-established mechanism of CAR T-cell therapy failure [43]. While this seems to occur in GBMs, a perhaps greater challenge is the baseline heterogeneity of surface antigen expression [9,19]. To address this challenge in other malignancies, a variety of multi-antigen targeting strategies have emerged [44]. In one approach, multiple independent CAR T-cells can be delivered either sequentially or as a mixed product [45]. Alternatively, a multivalent CAR in which a single construct is capable of binding to multiple target antigens can be engineered for unique functionality [44]. However, the functional optimization of these multivalent constructs can be more challenging, and incorporating multiple targeting domains increases the theoretical risk of off-tumor toxicity.

Several of these more sophisticated strategies to enable multi-antigen targeting and tackle the cellular heterogeneity of GBM have recently been described (see Figure 1). Choe and colleagues described the use of a synthetic Notch (synNotch) CAR circuit in which a more specific, but heterogeneous, GBM antigen—in this case, EGFRvIII—was detected by an engineered receptor in a “priming” step [10]. This priming step then induces the expression of a CAR with broader targeting ability—here, against EphA2 and IL13Ra2—which enabled antitumor activity. In combination, these elements function as a logical AND gate [10]. In another approach, Choi and colleagues developed a dual system in which an EGFRvIII-targeting CAR T-cell was modified to secrete a bispecific T-cell engager (BiTE) [9]. In this preclinical study, the BiTE used was targeted to both wild-type EGFR and CD3. In mouse xenograft models, this approach resulted in the elimination of EGFRvIII-heterogeneous GBMs. Intriguingly, this approach appeared to recruit bystander T-cells as well, raising the possibility that a broader antitumor immune response might be engaged using this strategy [9]. Beyond gliomas, numerous promising new technologies are being developed to address the challenge of heterogeneous tumor antigenicity, many of which could potentially be applied to the treatment of brain tumors as well [46,47,48]. In addition to reliable antigenicity, numerous challenges for CAR T-cell therapies common to brain tumors and other solid tumors remain, including microenvironmental suppression [41,42], T-cell exhaustion [48], and tumor apoptosis resistance [49].

Finally, a unique consideration for CAR T-cell therapies for brain tumors is the inherent toxicity that can result from an induced immunologic/inflammatory reaction within the central nervous system. The potential for CAR T-cell therapies to induce life-threatening neurotoxicity has been long-appreciated by practitioners in the field [37,50], but CNS-directed CAR T-cell therapies could induce additional neurologic damage simply by their activity on the healthy brain or the collateral effects of inflammation arising in the setting of tumor-directed therapy. In their current form, adoptive T-cell therapies targeting tumors that have infiltrated critical brain structures induce inflammation, leading to tumoral and peritumoral edema. This localized edema can alone induce neurologic symptoms. If edema leads to mass effect and compromises blood or cerebrospinal fluid circulation, ischemia or life-threatening increases in intracranial pressure can rapidly develop. Therefore, assembling multidisciplinary teams of clinicians and investigators with combined expertise in CAR T-cell therapies, neuro-oncology, and neurocritical care is a major barrier to clinical implementation. Expanded experience, including improved protocols for cell dosing and route of administration, may expand the accessibility of these treatments in the future. The inclusion of “kill-switch” systems in cell therapy products, such as inducible Caspase systems, may offer an additional layer of safety, particularly in early-stage trials where such clinical experience is limited [51,52]. However, because these systems are designed to completely inactivate the therapeutic cell product, they are necessarily a blunt instrument. The development of CARs with tunable activity, by chemical or other means, may enhance the neuro-oncologist’s control over CAR T-cell-induced neuroinflammation with greater precision, and improve the safety profile [53,54,55].

## 5. Conclusions

Adoptive cellular therapies, including CAR T-cells, have become standard therapies for several refractory hematologic malignancies and may play a major role in the future treatment of primary brain tumors, as highlighted by recent preliminary clinical trial results in glioma [15,35] and primary CNS lymphoma [56]. While responses have been heterogeneous so far, the reports of remarkable responses in patients with these devastating diseases suggest there is “signal in the noise” for these nascent neuro-oncological therapeutic approaches. Given the limitations of current treatments for many aggressive brain tumors, there is a clear and pressing need to continue developing these CAR T-cell therapies, and hopefully achieve the transformative impacts that have been demonstrated for other malignancies.

## Figures and Tables

**Table 1:** CAR T-cell trials in GBM registered with Clinicaltrials.gov (accessed on 18 February 2022). The following represents current clinical trials of CAR T-cells in adult GBMs registered with clinicaltrials.gov as of 18 February 2022. In some cases, details of the trial agent are unavailable.

Title	Clinical Trial Identifier	Age	Agent	Location	Status
Memory-Enriched T Cells in Treating Patients with Recurrent or Refractory Grade III-IV Glioma	NCT03389230	18–75 years	HER2-targeting, 4-1BB-CD3z CAR T-cells	City of Hope Medical Center Duarte, California, United States	Recruiting
IL13Ra2-CAR T Cells With or Without Nivolumab and Ipilimumab in Treating Patients With GBM	NCT04003649	≥18 years	IL13Ra2-4-1BB-CD3z CAR T-cells	City of Hope Medical Center Duarte, California, United States	Recruiting
Chimeric Antigen Receptor (CAR) T Cells with a Chlorotoxin Tumor-Targeting Domain for the Treatment of MPP2+ Recurrent or Progressive Glioblastoma	NCT04214392	≥18 years	Chlorotoxin (EQ)-CD28-CD3z CAR T-cells	City of Hope Medical Center Duarte, California, United States	Recruiting
CD147-CART Cells in Patients with Recurrent Malignant Glioma	NCT04045847	18–65 years	CD147-targeting CAR T-cells	National Translational Science Center for Molecular Medicine & Department of Cell Biology Xi’an, Shaanxi, China	Recruiting
NKG2D-based CAR T-cells Immunotherapy for Patient with r/r NKG2DL+ Solid Tumors	NCT05131763	18–75 years	NKG2DL-targeting, 4-1BB–CD3z CAR T-cells	Xunyang Changchun Shihua Hospital Jiujiang, Jiangxi, China	Recruiting
Pilot Study of B7-H3 CAR-T in Treating Patients with Recurrent and Refractory Glioblastoma	NCT04385173	18–75 years	B7-H3 targeting CAR T-cells	the Second Affiliated Hospital of Zhejiang University School of Medicine Hangzhou, Zhejiang, China	Recruiting
Safety and Efficacy Study of Anti-B7-H3 CAR-T Cell Therapy for Recurrent Glioblastoma	NCT05241392	18–75 years	B7-H3 targeting CAR T-cells	Beijing Tiantan Hospital Beijing, Beijing, China	Recruiting
Long-term Follow-up of Subjects Treated with CARv3-TEAM-E T Cells	NCT05024175	≥18 years	EGFRvIII-targeting, EGFR BiTE-secreting CAR T-cells	Massachusetts General Hospital, Dana Farber Cancer Institute Boston, Massachusetts, United States	Not yet recruiting
The Efficacy and Safety of Brain-Targeting Immune Cells (EGFRvIII-CAR T Cells) in Treating Patients with Leptomeningeal Disease From Glioblastoma	NCT05063682	≥18 years	EGFRvIII-4-1BB-CD3z CAR T-cells	Jyväskylä Central Hospital Jyväskylä, Finland University Of Oulu Oulu, Finland Apollo Hospital New Delhi, India	Active, not recruiting
CMV-specific Cytotoxic T Lymphocytes Expressing CAR Targeting HER2 in Patients With GBM	NCT01109095	N/A	HER2-CD28-CD3z, CMV-specific CAR T-cells	Houston Methodist Hospital Houston, Texas, United States Texas Children’s Hospital Houston, Texas, United States	Completed
CART-EGFRvIII + Pembrolizumab in GBM	NCT03726515	≥18 years	EGFRvIII-targeting, 4-1BB-CD3z CAR T-cells	Abramson Cancer Center of the University of Pennsylvania Philadelphia, Pennsylvania, United States	Completed

## Data Availability

No new data were created or analyzed in this study. Data sharing is not applicable to this article.
